# Changes in motor unit conduction velocity after unilateral lower‐limb suspension and active recovery are correlated with muscle ion channel gene expression

**DOI:** 10.1113/EP093065

**Published:** 2025-11-14

**Authors:** Giacomo Valli, Fabio Sarto, Francesco Negro, Elena Monti, Giuseppe Sirago, Matteo Paganini, Sandra Zampieri, Martino V. Franchi, Andrea Casolo, Julián Candia, Luigi Ferrucci, Marco V. Narici, Giuseppe De Vito

**Affiliations:** ^1^ Department of Clinical and Experimental Sciences University of Brescia Brescia Italy; ^2^ Department of Biomedical Sciences University of Padova Padova Italy; ^3^ Baxter Laboratory for Stem Cell Biology, Department of Microbiology and Immunology Stanford School of Medicine Stanford California USA; ^4^ Institute of Sport Sciences and Department of Biomedical Sciences University of Lausanne Lausanne Switzerland; ^5^ Department of Surgery, Oncology and Gastroenterology University of Padova Padova Italy; ^6^ Longitudinal Studies Section, Translational Gerontology Branch, National Institute of Aging National Institutes of Health Baltimore Maryland USA

**Keywords:** ion channel, mRNA sequencing, muscle fibre diameter, muscle unloading, neuromuscular impairment

## Abstract

The effects of muscle disuse on the propagation of action potentials along motor unit (MU) muscle fibres, a key process for effective muscle activation and force generation, remain poorly understood. The aim of this study was to investigate changes in action potential propagation and to identify biological factors influencing these changes following unilateral lower‐limb suspension (ULLS) and active recovery (AR). Eleven young males underwent 10 days of ULLS followed by 21 days of AR involving resistance exercise. Maximal force of the knee extensors (MVC), high‐density surface EMG recordings and muscle biopsies of the vastus lateralis muscle were collected before ULLS, after ULLS and after AR. EMG recordings collected during submaximal isometric contractions were decomposed to estimate single‐MU conduction velocity (CV). Biopsies were used to measure muscle fibre diameters via histochemical analysis and ion channel transcriptomic profiles via mRNA sequencing. The MVC declined by 29% after ULLS and returned to baseline after AR. MU CV decreased after ULLS and recovered fully, even exceeding baseline values after AR. Muscle fibre diameters did not change across the interventions and showed no correlation with MU CV. Conversely, a feature importance analysis revealed that mRNA expression levels of specific ion channel genes, particularly those involved in K^+^ transport, were correlated with MU CV at baseline and across the interventions. This study highlights the crucial role of K^+^ ion channels in influencing MU CV in humans, offering new insights into MU CV modulation and the mechanisms of changes in muscle force after disuse and active recovery.

## Abstract (highlights)

- **What is the central question of this study?**
  How does short‐term muscle disuse affect motor unit conduction velocity in humans, and which biological factors underlie these changes?
- **What is the main finding and its importance?**
  Motor unit conduction velocity decreases after muscle disuse and recovers fully after an active recovery period. This occurs independent of fibre size but is closely associated with ion channel gene expression, particularly of K⁺ channels. These findings highlight K⁺ ion channels as a key factor in regulating motor unit conduction velocity in humans and provide new molecular determinants of the changes in muscle force after disuse and recovery.

## 1 INTRODUCTION

Experimental models of muscle disuse are widely adopted to investigate the deleterious consequences of micro‐gravity or hospitalization‐induced inactivity (Qaisar et al., 2020) and to study their underlying mechanisms and potential countermeasures (Franchi et al., 2025; Michel et al., 2025). Disuse commonly leads to a reduction in both muscle mass and function, with the decline in muscle function significantly exceeding the loss of muscle mass (Campbell et al., 2019; Monti et al., 2021). This disparity suggests a pivotal role of neuromuscular factors in driving functional impairments (Piasecki, 2025).

Despite its crucial role in controlling force production, neuromuscular function has received relatively little attention in disuse research, particularly regarding motor unit (MU) properties. In fact, until recent years, only a few studies had investigated the changes in MU properties induced by muscle disuse. These studies observed a reduced MU discharge rate in small hand muscles after hand cast immobilization (Duchateau & Hainaut, 1990; Seki et al., 2007, 2001) and a reduced speed of action potential propagation along muscle fibres of single MUs in large leg muscles after bed rest (Cescon & Gazzoni, 2010). Although these previous studies built the foundation for understanding the impact of muscle disuse on neuromuscular function, several important questions remain unanswered.

Thanks to technological advancements in neuromuscular assessment techniques (Farina et al., 2016), recent studies have used state‐of‐the‐art intramuscular and high‐density surface electromyography (HDsEMG) to gain new insights into MU behaviour and neuromuscular adaptations during muscle disuse and recovery, with our research group having provided a substantial contribution to this field. For instance, a previous study performed on the same cohort investigated in the present manuscript (Valli et al., 2024b) shed light on the longstanding question of whether the reduction in MU discharge rate is observed preferentially in lower‐threshold MUs, as suggested by (Duchateau & Hainaut, 1990), or affects all MUs uniformly (Seki et al., 2001). Our findings, based on a large sample of Mus, showed that after 10 days of unilateral lower limb suspension (ULLS), the reduction in discharge rate is indeed specific to lower‐threshold MUs, suggesting that this selective decrement of neural drive might lead the shift in fibre type phenotype (from slow‐ to fast‐twitch) that has been reported after longer periods of disuse (Bodine, 2013). Focusing instead on MU central properties, we have also shown that neuromodulation decreases following ULLS, which mighty explain, in part, the diminished neural drive to the muscle (Martino et al., 2024). In contrast, investigations performed with intramuscular EMG demonstrated that the neuromuscular junction is functionally resilient to 10–15 days of muscle unloading (Sarto et al., 2022) or immobilization (Inns et al., 2022) and, therefore, it remains capable of conveying the neural discharge reliably to the muscle fibres within this time frame.

In summary, the neuromuscular consequences of disuse have been characterized at the spinal, motoneuronal and neuromuscular junction levels. However, modifications at the final component of the neuromuscular system, the muscle unit (i.e., the group of muscle fibres innervated by a single motor neuron) (Heckman & Enoka, 2004), remain to be elucidated fully.

The aim of this study was to clarify the modifications occurring at the muscular component of the MU following 10 days of ULLS and subsequent active recovery (AR) and to identify possible factors involved in these adaptations. To achieve this, we used HDsEMG recordings to study the conduction velocity (CV) of single MU action potentials (MUAPs) and muscle biopsies to investigate muscle fibre diameters and ion channel mRNA expression. We focused on MU CV, a fundamental marker of MU contractile properties, which represents the speed at which an action potential travels along the muscle fibres of a single MU (Andreassen & Arendt‐Nielsen, 1987). From a biophysical perspective, it has been observed that the CV of electrically evoked action potentials is correlated with vastus lateralis muscle fibre size (Methenitis et al., 2016), although the molecular determinants of MU CV in humans remain poorly understood. To solve this, we combined the analysis of muscle fibre diameters with analysis of the mRNA expression of their constituent ion channels, because these channels play a crucial role in the generation and propagation of action potentials and might significantly influence MU CV and its changes (Jurkat‐Rott & Lehmann‐Horn, 2004).

We hypothesized that MU CV would decrease after ULLS and recover after AR. We further posited that these changes in MU CV could be explained by alterations in muscle fibre diameters and/or ion channel mRNA expression.

For clarity, this study is part of a broader research effort conducted by our group to clarify the neuromuscular adaptations to disuse and recovery. Previous publications based on the same experimental cohort have focused on central MU properties (Martino et al., 2024; Valli et al., 2024b), neuromuscular transmission (Sarto et al., 2022) or molecular patterns (Franchi et al., 2025; Sirago et al., 2023), whereas the present manuscript specifically investigates changes occurring at the muscular component of the MU. Crucially, the breadth of investigations and the richness of the available dataset are key to the novelty of this study, which integrates HDsEMG, muscle biopsy and molecular approaches. This design enabled analyses that would not be feasible in more narrowly focused EMG studies and offers extremely novel insights into MU adaptations to muscle disuse and recovery.

## 2 MATERIALS AND METHODS

This study was approved by the Ethics Committee of the Department of Biomedical Sciences of the University of Padova (Italy) with reference number HEC‐DSB/01‐18. Professor Rosario Rizzuto, Director of the Department of Biomedical Sciences, was responsible for research governance. The study was conducted in accordance with the standards set by the latest revision of the *Declaration of Helsinki*. Participants were informed about all the experimental procedures through an interview and information sheets. Their eligibility was determined after a thorough review of their medical history. Volunteers were enrolled in the study after signing a written consent form, with the option to withdraw at any time.

Table 1 provides a summary of parallel works conducted with the same cohort and how these complement each other. For a detailed explanation of the unloading and active recovery models and biological data extraction, the reader can refer to Sarto et al. (2022), and for a detailed explanation of the HDsEMG data collection to Valli et al. (2024b).

**TABLE 1:** Related publications from our group on limb suspension and active recovery.

Publication	Title	Topic
Sarto et al. (2022)	Effects of short‐term unloading and active recovery on human motor unit properties, neuromuscular junction transmission and transcriptomic profile	Stability of the neuromuscular junction (electrophysiology and biology)
Sirago et al. (2023)	Upregulation of sarcolemmal hemichannels and inflammatory transcripts with neuromuscular junction instability during lower limb unloading in humans	Changes in sarcolemmal hemichannels, inflammation and neuromuscular junction stability (biology)
Valli et al. (2024b)	Lower limb suspension induces threshold‐specific alterations of motor units properties that are reversed by active recovery.	Threshold‐specific adaptations of central motor unit properties (electrophysiology)
Martino et al. (2024)	Neuromodulatory contribution to muscle force production after short‐term unloading and active recovery	Motoneuron excitability (electrophysiology)
Franchi et al. (2025)	Previous short‐term disuse dictates muscle gene expression and physiological adaptations to subsequent resistance exercise	Muscle structural, metabolic and gene expression adaptations (biology)

### 2.1 Participants and experimental protocol

Twelve healthy, recreationally active young male adults [age, 22.1 (2.9) years; height, 1.78 (0.03) m; body mass, 72.1 (7.1) kg] volunteered for this study. To minimize the risk of deep venous thrombosis associated with ULLS (Bleeker et al., 2004), only male participants were included, because among young individuals the absolute risk of first venous thrombosis is higher in females (Roach et al., 2014). The inclusion criteria were as follows: age between 18 and 35 years; a body mass index of 20–28 kg/m^2^; and engagement in recreational physical activities one to three times per week (self‐reported). Exclusion criteria included a sedentary lifestyle, participation in recreational physical activities more than three times per week, smoking, a history of deep venous thrombosis, and any other conditions that might prevent safe participation in the study.

**FIGURE 1:**
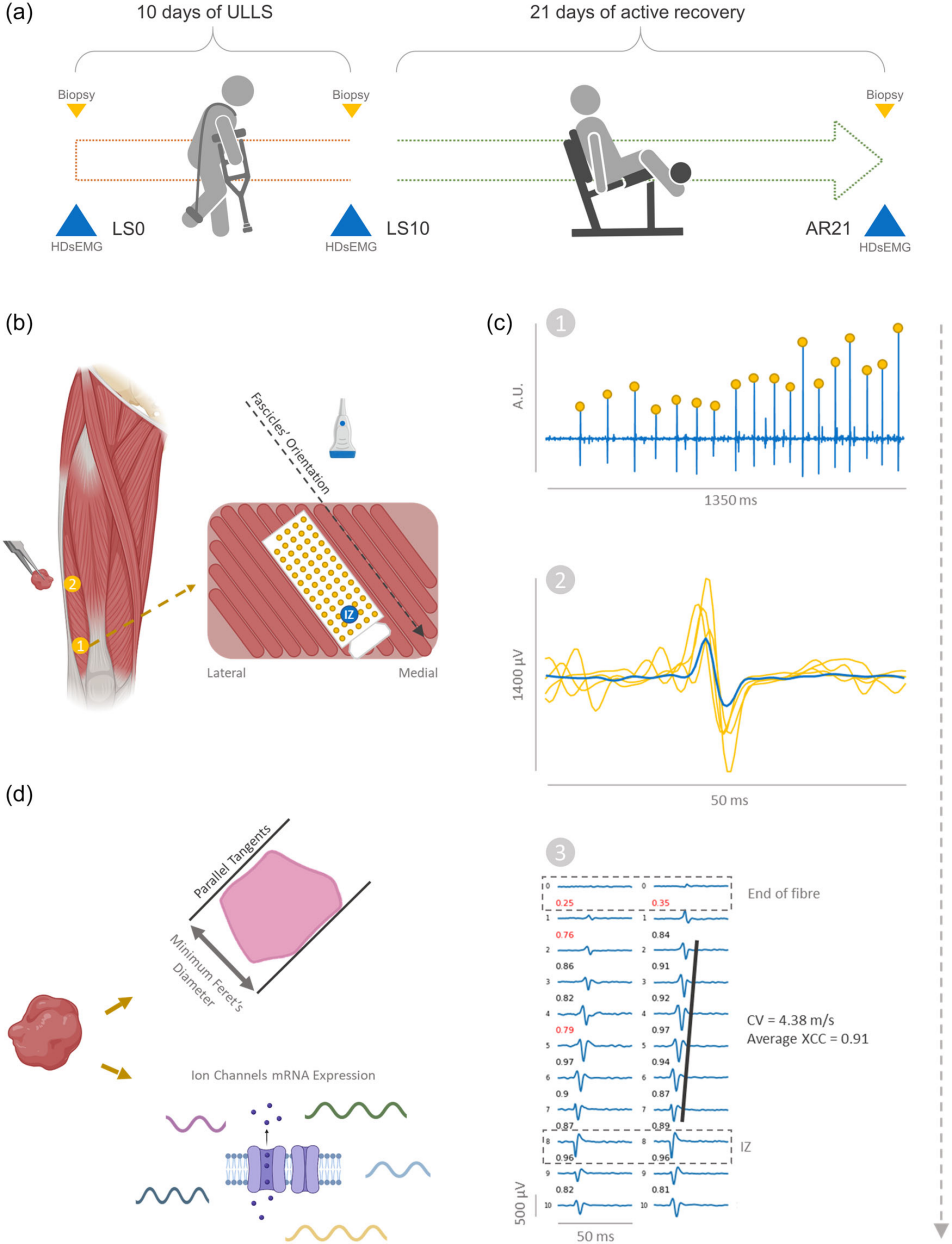
Schematic representation of the study design (a) and procedures of data collection and analysis (b–d). (a) Data were collected at baseline (day 0 of limb suspension, LS0), after 10 days (LS10) of unilateral lower‐limb suspension (ULLS) and after 21 days of active recovery (AR21). (b) The expected location of the used innervation zones (IZ) in the vastus lateralis muscle. The central zone (around IZ number 2), was used for biopsy collection, whereas the distal zone (around IZ number 1) was used for high‐density surface electromyography (HDsEMG) recordings. Also shown in (b) is the orientation of the recording grid with respect to the muscle fascicles and the IZ. (c) The procedure used for the extraction of motor unit action potentials (MUAPs) and for MU conduction velocity (CV) estimation is depicted. Specifically, the time of firing of each MU (c1) was used to trigger and average the MUAPs in each channel of the grid (c2), and the best‐propagating channels were selected for MU CV estimation via a maximum likelihood algorithm (c3). (d) Muscle biopsies were used for estimation of the minimum Feret's diameter and for the measurement of the ion channel gene expression via bulk RNA sequencing. The cross‐correlation coefficient (XCC) is the measure of similarity between the MUs action potential shape.

Data collection was performed at baseline (day 0 of limb suspension, LS0), after 10 days of ULLS (LS10) and after 21 days of active recovery (AR21) (Figure 1a). All the participants visited the laboratory before the LS0 data collection to familiarize themselves with the ULLS procedures and with the isometric muscle contractions. At LS10, participants were tested immediately after ending limb suspension and were allowed to warm up only before the MVC test. At AR21, tests were conducted ∼72 h after the final exercise session to avoid potential muscle fatigue.

Participants were instructed to avoid intense exercise, coffee and alcohol for 24 h before any data collection. All tests were performed at the same time of day for each participant.

### 2.2 Intervention

#### 2.2.1 Unilateral lower‐limb suspension

The dominant lower limb (the kicking leg, which was the right leg for all participants) was suspended in a slightly flexed position (15°–20° of knee flexion) for 10 days. To ensure that the suspended limb did not touch the ground while moving, the left lower limb was fitted with an elevated shoe (50 mm sole) (Berg et al., 1991). Throughout the ULLS period, participants walked exclusively using crutches and avoided any weight‐bearing on the unloaded limb. Participants were instructed to avoid any active contraction of muscles of the suspended limb. However, they were allowed to perform passive, non‐weight‐bearing ankle exercises (Bleeker et al., 2004) to promote blood circulation and reduce the risk of deep venous thrombosis. Participants were recommended to wear elastic compression socks on the suspended lower limb during ULLS. Compliance of participants was evaluated through daily calls and messages.

#### 2.2.2 Active recovery

The AR phase began ∼72 h after the suspension period and lasted for 21 days. During the AR phase, participants performed unilateral resistance exercises three times per week, with ≥24 h of rest between sessions. Each session included three sets of 10 repetitions of leg‐press and leg‐extension exercises at 70% of the estimated one‐repetition maximum, following a warm‐up at 30% of one‐repetition maximum. Exercises were performed from full knee extension to ∼90° flexion, with a time under tension of ∼2 s in both the concentric and eccentric phases. A 2 min rest was observed between sets.

The one‐repetition maximum was estimated from the heaviest load that participants could lift for four to six repetitions during the first session (Brzycki, 1993) and was re‐evaluated weekly to adjust the load. This approach was chosen owing to participants’ lack of prior resistance training and the recent unloading of their lower limb.

### 2.3 Measurements

#### 2.3.1 High‐density surface electromyography grid placement

The HDsEMG signals were recorded from the distal portion of the vastus lateralis muscle (Figure 1b) using an adhesive grid of 64 electrodes (five columns and 13 rows with 8 mm interelectrode distance; GR08MM1305, OT Bioelettronica, Torino, Italy) filled with conductive cream (Ac cream, OT Bioelettronica).

A meticulous two‐step approach was adopted to ensure the precise alignment of columns of the grid with respect to the muscle fibres and to maintain a standardized position of the grid with respect to the muscle innervation zone, because these are fundamental prerequisites for reliable MU CV estimation (Valli et al., 2024a). First, the innervation zone was detected as the location in which the largest muscle twitch was evoked in response to low‐intensity percutaneous electrical stimulation using a pen electrode (Botter et al., 2011). Given that vastus lateralis presents different innervation zones, we focused on the most distal one, which is generally located between 35% and 20% of femur length (measured from the distal end) and provides better MU decomposition (Botter et al., 2011; Figure 1b). Stimulations were induced using an electrical stimulator (DS7AH, Digitimer Ltd, Welwyn Garden City, UK), with an electrical current set at 16 mA (400 V; pulse width, 50 µs). After the innervation zone was identified, the current was reduced to 8–10 mA to narrow the identified location. Second, the orientation of muscle fascicles over the innervation zone was detected using B‐mode ultrasound recordings (Mylab70, Esaote, Genoa, Italy) (Hug et al., 2021), and it was marked on the skin with a permanent marker.

Subsequently, the grid was placed following the orientation of muscle fascicles and above the innervation zone (Barbero et al., 2012; Botter et al., 2011). Specifically, the innervation zone corresponded to the central electrodes of the last two to three rows of the grid. Please refer to Figure 1b for a visual representation.

The ultrasound system was also used to detect muscle borders accurately and to avoid the placement of the grid across adjacent muscles.

Before positioning the grid, the skin was carefully shaved, cleaned with 70% ethanol, then treated with abrasive–conductive paste (Spes medica, Salerno, Italy). Two reference electrodes were placed on the malleolus and patella bones.

After the recordings, the grid border was marked with a permanent marker on the skin. The operator emphasized these markings at each participant meeting to ensure that exact placement of the grid could be reproduced in the subsequent data collection points (Casolo et al., 2020).

#### 2.3.2 High‐density surface electromyography recordings

Maximum voluntary contraction (MVC) of the knee extensor muscles was assessed at 90° knee angle using a custom‐made knee dynamometer fitted with a load cell (RS 206‐0290), which was attached above the ankle using straps. The participant's back was supported in an upright position, with a resulting hip angle of 90°. The hip was stabilized to the table with adjustable straps to limit compensation (Monti et al., 2021). Participants were instructed to perform the task during the familiarization session and, after a standardized warm‐up, were asked to ‘push as hard as possible’ by extending the dominant leg against the load cell, then to maintain the contraction for 3–4 s. Loud verbal encouragement and visual feedback were provided. The test was repeated three times with 60 s of rest, and the contraction with the maximum value was used to determine the target forces used for HDsEMG recordings.

The HDsEMG signal was recorded during submaximal isometric trapezoidal contractions performed at 10%, 25% and 50% LS0 MVC (i.e., the baseline MVC value was used as a reference for all the submaximal contractions performed at each data collection point). Each contraction consisted of a ramp‐up phase to the submaximal target force level, which was then maintained during a steady‐state phase, followed by a ramp‐down phase back to baseline. All the contractions had a total duration of 30 s. The ramp‐up and ramp‐down phases were performed with a linear force increase/decrease set at 5% MVC/s (Del Vecchio et al., 2019), and the duration of the steady‐state phase was adjusted accordingly (i.e., at 50% MVC the ramps lasted 10 s each and the steady‐state 10 s, which adds up to 30 s of total contraction). Each intensity level of the trapezoidal contractions was repeated twice, in random order, with 60 s of rest in between. Participants received real‐time visual feedback of the force produced and were instructed to match it as precisely as possible during the familiarization session.

The EMG and force signals were sampled at 2048 Hz with the EMG‐Quattrocento (OT Bioelettronica). The EMG signal was recorded in monopolar configuration, amplified (×150) and bandpass filtered (10–500 Hz) at the source. Force was recorded synchronously with the EMG signal, and the offset was removed before starting the recording.

#### 2.3.3 Motor unit detection and conduction velocity estimation

The force signal was low‐pass filtered using a fourth‐order, zero‐lag Butterworth filter with a 15 Hz cut‐off. The HDsEMG signal was bandpass filtered between 20 and 500 Hz with a second‐order, zero‐lag Butterworth filter and decomposed to obtain the discharge pattern of individual MUs with convolutive blind source separation (Figure 1c1; Negro et al., 2016). The majority of the MUs included in this study are the same as in our previous publication (Valli et al., 2024b). However, the decomposition of some contractions with a limited number of detected MUs was repeated, with the aim of maximizing the number of MUs suitable for CV analysis. Specifically, particular attention was dedicated to the removal of noisy channels, and the decomposition was run until the residual activity index reached zero, rather than up to a fixed number of iterations. This additional decomposition also allowed for the inclusion of one more participant in the analyses in comparison to our previous publication (Valli et al., 2024b). After decomposition, the discharge pattern was inspected and edited manually by an experienced operator following recent guidelines (Del Vecchio et al., 2020; Martinez‐Valdes et al., 2023), and only MUs with a pulse‐to‐noise ratio of ≥28 were maintained for further analyses (Holobar et al., 2014).

The MUs detected during the two contractions performed at the same intensity level during the same data collection point were pooled and analysed together after the removal of duplicated Mus, as previously explained (Valli et al., 2024b). Briefly, the MUs detected in both contractions were matched via comparison of their MUAPs representation, and those with a cross‐correlation coefficient (XCC) of >0.9 where classified as duplicates (Maathuis et al., 2008; Martinez‐Valdes et al., 2017). Of the two duplicates, the MU with the lowest pulse‐to‐noise ratio was removed. This approach allowed us to obtain a broader and more representative number of MUs to include in the following analysis, which is an important aspect, especially considering the strict criteria used for the selection of MUs suitable for CV analysis.

For MU CV estimation, representation of the MUAPs across the channels of the grid was obtained from spike‐triggered averaging of the double‐differential derivation of the EMG signal along the direction of the muscle fibres (Figure 1c2; Casolo et al., 2020). This spatial filtering is important to enhance the representation of MUAPs propagation, because it decreases the presence of non‐propagating components and attenuates the end‐of‐fibre effect (Gallina et al., 2022). The spike‐triggered averaging was performed using the first 50 MU discharges (Martinez‐Valdes et al., 2018) and with a 50 ms window (Valli et al., 2024a, 2025). The channels used for MU CV estimation were selected manually by an experienced operator using the following inclusion criteria: (1) a clear propagation of the action potential; (2) an XCC between adjacent channels of >0.8; and (3) no innervation zone or end‐of‐fibre effect (Figure 1c3). If multiple columns presented similarly suitable MUAPs, the selection prioritized the column with the highest average XCC between adjacent channels (Valli et al., 2024a). On the selected channels, MU CV was calculated using the maximum likelihood estimation of delay previously proposed by Farina et al. (2002, 2000). Although this maximum likelihood algorithm can, theoretically, work with a minimum of two signals, we always selected the greatest possible number of channels in order to maximize the accuracy of the estimation.

For the scope of this study, we investigated only the total pool of decomposed MUs, without applying any longitudinal tracking procedure (Martinez‐Valdes et al., 2017). This was preferred because MU tracking across three time points, with ULLS and AR between them, greatly reduces the number of MUs suitable for analysis (to <20% of the total pool; Valli et al., 2024b), which would be reduced further after applying the inclusion criteria required for accurate MU CV estimation. Therefore, channel selection for MU CV estimation was performed independently for each MU at each data collection point, with the operator blind to the condition.

For correlation analyses, the mean MU CV values for participants were obtained by averaging the mean MU CV at the three contraction intensities [i.e., mean MU CV = (mean MU CV at 10% MVC + mean MU CV at 25% MVC + mean MU CV at 50% MVC)/3]. This method was chosen over a global average of all MU CV values, because it avoids bias from differences in the number of MUs detected at each contraction intensity (Casolo et al., 2023). Throughout this manuscript, ‘mean MU CV’ refers to this calculated average. Mean CV was used for all the correlation analyses except for the correlation between MU recruitment threshold (RT) and CV, as explained in section 2.4.

For the MUs where CV could be estimated, the relative MU RT was also calculated [i.e., the relative (% MVC) force level corresponding to the first discharge time].

MU decomposition was performed with custom Matlab scripts (R2023a; The Mathworks Inc., Natick, MA, USA), and all the analyses were performed with the *openhdemg* v.0.1.0 library (Valli et al., 2024a) in Python (v.3.11.6, Python Software Foundation, USA).

#### 2.3.4 Muscle biopsy collection and muscle fibre diameter estimation

From each participant, a muscle biopsy of ∼150 mg was collected from the vastus lateralis muscle using a Weil–Blakesley conchotome (Gebrüder Zepf Medizintechnik GmbH & Co. KG, Dürbheim, Germany). The three biopsies were performed at 2 cm from the central innervation zone and with a distance between each other of ∼2–3 cm to avoid effects of presampling (Sarto et al., 2022). With this set‐up, the bandage of the biopsy (and consecutive swelling) would not affect HDsEMG recordings, which were performed on the distal innervation zone. Furthermore, given that the longitudinal position of the biopsy site did not affect biological properties (including muscle fibre diameters) in the vastus lateralis (Horwath et al., 2021), the use of different sites for the collection of biopsies and HDsEMG recordings should not introduce any bias into the relationship between biological and electrophysiological parameters.

Within 3–5 min from data collection, a portion (∼30 mg) of the muscle was cleaned of connective and adipose tissue, embedded in an oriented manner in optimal cutting temperature (OCT) compound, frozen in isopentane, and stored at −80°C for histochemical analysis. Cryosections were made using a manual cryostat (Leica CM1850; Leica Microsystems, Wetzlar, Germany), producing sections 10 µm thick. To evaluate muscle fibre diameter depending on fibre type, myofibrillar ATPase staining was performed on serial cross‐sections, optimizing the protocol of Brooke & Kaiser (1970) with a pre‐incubation step in 0.1 m potassium acetate buffer at pH 4.35, for 10 min at room temperature. After washes in 0.1 m Tris–HCl and 18 mm CaCl_2_ at pH 7.8, sections were incubated in 90 mm CaCl_2_ and 0.1 m sodium barbiturate buffer containing ATP with pH adjusted to 9.4, for 45 min at 37°C. The slides where then incubated in 2% CoCl_2_ at room temperature, and the staining was developed by 5% ammonium sulphide solution. Sections were then dehydrated, cleared in xylene and mounted in Canada balsam. By this protocol, slow‐twitch muscle fibres (those possessing a higher ATPase activity) were visualized as dark, whereas fast‐type fibres (or those possessing a low ATPase activity) were lightly stained. Stained sections were then observed under a Zeiss microscope, and images of each entire section were captured at ×20 magnification using a Leica DC 300F camera connected to the microscope. Muscle morphometry was assessed on a photomerge of single images automated by Adobe Photoshop.

Given that estimation of muscle fibre diameter can be affected by the orientation of the sectioning angle or by kinked muscle fibres (Dubowitz et al., 2013), we opted to estimate fibre diameters by measuring the minimum Feret diameter, defined as the minimum distance of parallel tangents at opposing borders of the muscle fibre. This approach can minimize the bias introduced by the variable cutting of the sections (Briguet et al., 2004). The minimum Feret diameter of each muscle fibre was measured manually across all visible fibres (∼200–400 per section) using the software ImageJ v.1.52v (Schindelin et al., 2015).

#### 2.3.5 Ion channel mRNA expression

Full description of mRNA sequencing data extraction, starting from the biopsies, can be found in the paper by Sarto et al. (2022). For the scope of this study, mRNA sequencing raw counts have been normalized using the R package DESeq2 v.4.4.1 (Love et al., 2014) called from Python via the rpy2 package v.3.5.16 and associated with the corresponding metadata (i.e., participant and data collection point). Of all the available genes, we extracted those present in skeletal muscle ion channels based on the study by Jurkat‐Rott and Lehmann‐Horn (2004). Of the 40 identified ion channels, 35 genes were retained for analysis, because 5 were not detectable in the dataset.

For clarity, differential expression of these genes has already been reported by Sarto et al. (2022).

### 2.4 Statistical analysis

For MVC, muscle fibre diameters (slow‐twitch fibre, fast‐twitch fibre and combined fibre diameters) and mRNA expression of selected ion channel genes (*KCNJ2‐AS1*, *KCNN2*, *KCNN3* and *SCN4A*) variables, normality of the distribution was assessed by the Shapiro–Wilk test. Given that the normality assumption was satisfied, a one‐way repeated‐measures ANOVA was used. For all ANOVAs, sphericity was tested with Mauchly's test, and if the assumption of sphericity was violated, the Greenhouse–Geisser correction was applied. If the ANOVA was significant, the *post hoc* pairwise *t*‐tests with Holm correction were used to determine whether differences among the time points were present. Partial eta squared (η_p_^2^) was also estimated to evaluate the effect size for ANOVAs.

The MU CV was analysed using a linear mixed‐effects model, because multiple MUs were detected from each participant (Yu et al., 2022). The normality of the model residuals was assessed through visual inspection of the Q–Q plot and histogram. Given that normality of the residuals was confirmed, the linear model was applied, with ‘time’ and ‘intensity’ as factors and with the ‘participant’ as a cluster variable. *Post hoc* comparisons were performed with the Holm correction.

A linear mixed‐effects model was also applied to investigate the relationship between MU RT and CV and to determine whether this relationship varied across the different data collection points. Specifically, the latter was assessed by comparing changes in the slopes and intercepts of the regression lines for mean MU RT and CV values at each data collection point. For this analysis, we used the mean RT and CV values at each contraction intensity (i.e., three values per participant, one each at 10%, 25% and 50% of MVC) instead of the previously described overall mean RT and CV. This choice helped to minimize overfitting in the correlation analysis and to capture a broader range of values. Additionally, this approach yielded similar correlation values to the mean within‐participant correlation method used by Casolo et al. (2023) (data not shown), while allowing for a simpler model to compare slopes and intercepts across data collection points.

Repeated‐measures correlation (Bakdash & Marusich, 2017) was used to determine whether changes in mean MU CV across the three data collection points were associated with changes in MVC, combined muscle fibre diameters and ion channel mRNA expression. For this analysis, average values for each participant MU CV were used as a representation of the clustered values, in order to reduce the variability of the model, as previously suggested (Valli et al., 2024b).

Correlation analyses for variables of interest were performed using the Pearson correlation coefficient and *p*‐value.

A feature importance analysis was conducted to identify which predictive variables (i.e., muscle fibre diameters and ion channel mRNA expression) had the most significant impact on the prediction of mean MU CV (the outcome variable). This analysis was performed using a random forest regressor model (Breiman, 2001) trained with leave‐one‐out cross‐validation, which maximizes the training data available (in each iteration, the model was trained on all observations except one, optimizing parameters based on the remaining data). The importance of each predictive feature was assessed by its contribution to the predictive accuracy of the model. Specifically, in each tree of the forest, the algorithm chooses splits that reduce the prediction error, and the amount of error reduction achieved is attributed to the feature used at that split. By summing these contributions across all splits and all trees, the model derives an importance score for each feature, reflecting how strongly it improves predictive accuracy. Results from all iterations were aggregated to calculate the mean importance score for each feature across all folds. This analysis estimates the relative significance of each variable in explaining mean MU CV. Feature importance analysis was conducted both including all data collection points (to estimate the importance of the predicting variables across the interventions) or including only LS0 (to estimate the importance of the predicting variables at baseline).

The mixed model for MU CV was computed with jamovi v.2.2.2 (Sydney, NSW, Australia; R language), whereas all the other statistical analyses were done using Python (v.3.11.6, Python Software Foundation, USA), with the statsmodels v.0.14.1 (Seabold & Perktold, 2010), scikit‐learn v.1.5.0 (Pedregosa et al., 2011) and pingouin v.0.5.4 (Vallat, 2018) libraries. Statistical significance was accepted at *p *< 0.05. The results are reported and plotted as the mean (SEM) for linear models and are reported and plotted as the mean (SD) for the other analyses.

## 3 RESULTS

### 3.1 Participants

Of 12 participants, one dropped out after baseline measures for personal reasons. Eleven participants completed the study successfully, without any adverse event. We were able to perform MU analysis on all the 11 participants at all the data collection points. However, two participants opted not to undergo muscle biopsies at AR21, and one participant was excluded from the analysis of muscle fibre diameters at LS10 owing to an insufficient amount of muscle sample.

### 3.2 Maximum voluntary contraction

MVC decreased at LS10 and returned to LS0 values at AR21 (main effect of time: *p *< 0.0001, η_p_^2^ = 0.8620). More details are presented in Table 2.

**TABLE 2:** Changes in maximum voluntary contraction across the intervention.

Data collection point	MVC (N), mean ± SD	Change (%) from LS0	*p*‐value versus LS0
LS0	795.96 ± 112.83	–	–
LS10	563.37 ± 101.01	−29.23	<0.0001
AR21	791.07 ± 107.06	−0.61	0.8342

Abbreviations: AR21, after 21 days of active recovery; LS0, day 0 of limb suspension; LS10, after 10 days of unilateral lower‐limb suspension; MVC, maximum voluntary contraction.

### 3.3 Motor unit decomposition and conduction velocity analysis

A total of 1542 unique MUs (654 at 10%, 577 at 25% and 311 at 50% of MVC) were identified. Of these, 939 MUs (60.89% of the total pool; 332 at 10%, 383 at 25% and 224 at 50% of MVC) were suitable for MU CV analysis. This resulted in an average number of MUs per participant and per data collection point of 10.37 (5.28) at 10%, 11.96 (6.00) at 25% and 7.47 (3.89) at 50% of MVC.

On average, MU CV has been estimated on 4.00 (0.84) channels for each MU, with a very high XCC between the selected channels of 0.95 (0.03) at LS0, 0.96 (0.02) at LS10 and 0.95 (0.03) at AR21.

**FIGURE 2:**
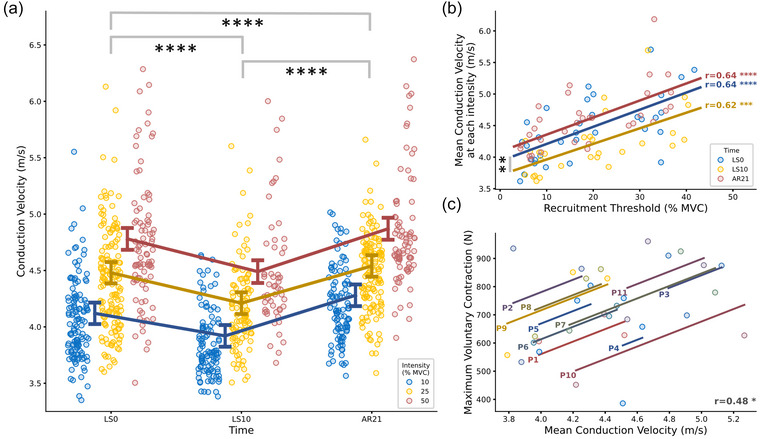
Analysis of motor unit (MU) conduction velocity (CV) and its association with MU recruitment threshold (RT) and maximum voluntary contraction (MVC). (a) Swarm plot representing MU CV at the three data collection points. Different intensities of contraction (i.e., 10%, 25% and 50% MVC) are represented by different colours. Single MUs are represented by dots. Summary data are presented as the mean ± SEM, and the direction of the changes is highlighted by a connecting line. Significant differences between the data collection points have been marked. (b) Correlation analysis between mean relative MU recruitment threshold (RT) and CV. The correlation was performed at each data collection point (represented by different colours) on mean MU RT and CV values. Mean values were estimated for each participant at each contraction intensity (i.e., three values per participant, per data collection point). Significant differences between the intercept of the regression lines are marked. (c) Common within‐individual association between MU CV and MVC across the data collection points. Participants are represented by different colours. Abbreviations: AR21, after 21 days of active recovery; LS0, day 0 of limb suspension; LS10, after 10 days of unilateral lower‐limb suspension; MVC, maximum voluntary contraction. Significance levels are as follows: *p < 0.05, **p < 0.01, ***p < 0.001 and ****p < 0.0001.

MU CV (Figure 2a) showed a significant effect of time (*p *< 0.0001). MU CV was reduced at LS10 compared with LS0 (*p *< 0.0001), whereas it increased at AR21 up to and exceeding the LS0 values (*p *< 0.0001). All the contraction intensities exhibited similar changes (no time × intensity effect, *p *= 0.4103), although MU CV differed between contraction intensities (intensity effect, *p *< 0.0001). Estimated MU CV values and *post hoc* tests are presented in Table 3.

**TABLE 3:** Motor unit conduction velocity estimated means and *post hoc* tests for significant effects.

Estimated marginal means: Time				
Time	Mean	SEM		
LS0	4.46	0.0926		
LS10	4.21	0.0935		
AR21	4.57	0.0928		
**Estimated marginal means: Intensity**				
Intensity	Mean	SEM		
10	4.11	0.0927		
25	4.41	0.0926		
50	4.71	0.0937		
**Estimated marginal means: Intensity:Time**				
Intensity	Time	Mean	SEM	
10	LS0	4.12	0.0952	
25	LS0	4.48	0.0946	
50	LS0	4.78	0.0969	
10	LS10	3.92	0.0962	
25	LS10	4.21	0.0963	
50	LS10	4.49	0.1007	
10	AR21	4.28	0.0962	
25	AR21	4.54	0.0951	
50	AR21	4.87	0.0975	
***Post hoc* comparisons: Time**				
Time	Time	Difference	SEM	*p* Holm
AR21	LS0	0.103	0.0246	< 0.0001
LS10	LS0	−0.255	0.0264	< 0.0001
AR21	LS10	0.358	0.0272	< 0.0001
***Post hoc* comparisons: Intensity**				
Intensity	Intensity	Difference	SEM	*p* Holm
25	10	0.304	0.0237	< 0.0001
50	10	0.606	0.0281	< 0.0001
50	25	0.303	0.027	< 0.0001

*Note*: Summary statistics of MU CV were analysed with linear mixed models. Values are expressed in metres per second. Abbreviations: AR21, after 21 days of active recovery; LS0, day 0 of limb suspension; LS10, after 10 days of ULLS; MU CV, motor unit conduction velocity.

MU RT and CV were significantly correlated at each data collection point (*r* = 0.64, *p *< 0.0001 at LS0; *r* = 0.62, *p *= 0.0004 at LS10; and *r* = 0.64, *p *< 0.0001 at AR21; Figure 2b). No significant differences were detected between the slopes of the regression lines (*p *= 0.7714 for LS0 vs. LS10; and *p *= 0.9882 for LS0 vs. AR21). The intercepts of the regression lines differed between LS0 and LS10 (*p *= 0.0059) but not between LS0 and AR21 (*p *= 0.1136).

Changes across data collection points in MU CV were significantly correlated with changes in MVC (*r* = 0.48, *p *= 0.0236; Figure 2c).

### 3.4 Muscle fibre diameters

**FIGURE 3:**
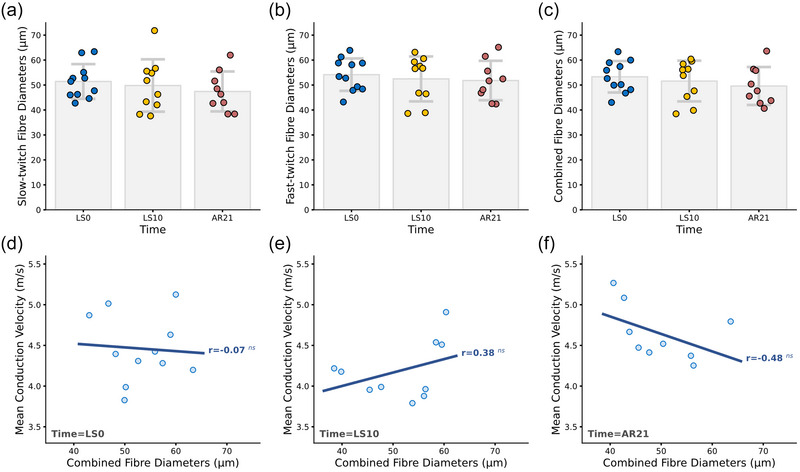
Analysis of muscle fibre diameters and their association with MU CV. Scatter plot and box plot representing individual values and mean ± SD of slow‐twitch (a), fast‐twitch (b) and combined (c) fibre diameters at each data collection point. (d–f) Scatter plots showing the correlation between total fibre diameters and mean MU CV at each data collection point. Abbreviations: AR21, after 21 days of active recovery; LS0, day 0 of limb suspension; LS10, after 10 days of unilateral lower‐limb suspension; MU CV, motor unit conduction velocity. Significance levels are as follows: *^ns^p* > 0.05.

Muscle fibre diameters did not change throughout the intervention, regardless of fibre type. Slow‐twitch fibres had a diameter of 51.43 (6.95) µm at LS0, 49.81 (10.49) µm at LS10 and 47.43 (7.10) µm at AR21 (*p *= 0.3102, η_p_^2 ^= 0.1540; Figure 3a). Fast‐twitch fibres had a diameter of 54.17 (6.50) µm at LS0, 52.46 (9.01) µm at LS10 and 51.81 (7.90) µm at AR21 (*p *= 0.7106, η_p_^2 ^= 0.0476; Figure 3b). When including both slow‐ and fast‐twitch fibres, the combined fibres had a diameter of 53.30 (6.30) µm at LS0, 51.60 (8.16) µm at LS10 and 49.61 (7.60) µm at AR21 (*p *= 0.3953, η_p_^2 ^= 0.1242; Figure 3c).

There was no significant correlation between total fibre diameters and mean MU CV values at each data collection point, as demonstrated by the correlation analyses (*r* = −0.07, *p *= 0.8367 at LS0; *r* = 0.38, *p *= 0.2812 at LS10; and *r* = −0.48, *p *= 0.1928 at AR21; Figure 3d–f).

Likewise, there was no significant correlation between changes in total fibre diameters and mean MU CV values across the data collection points, as demonstrated by repeated‐measures correlation analysis (*r* = 0.01, *p *= 0.9675; not shown in figure).

### 3.5 Ion channel mRNA expression

**FIGURE 4:**
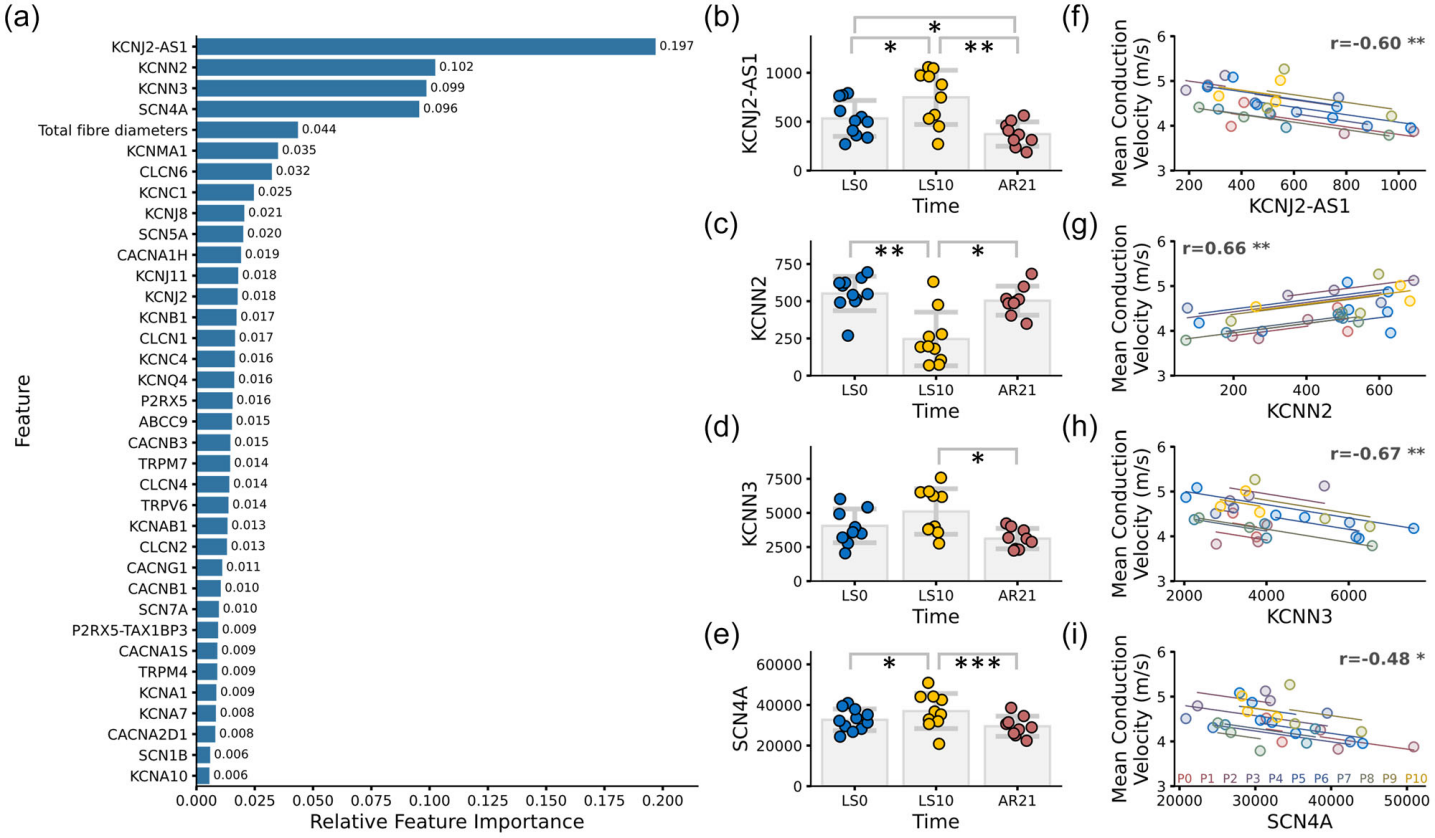
Analysis of ion channel mRNA expression levels and their association with MU CV across the data collection points. (a) Bar plot representing the relative importance of each feature (ion channel genes and total muscle fibre diameters) for the prediction of MU CV. (b–e) Scatter plot and box plot representing individual values and the mean ± SD of *KCNJ2‐AS1* (b), *KCNN2* (c), *KCNN3* (d) and *SCN4A* (e) mRNA expression levels (normalized reads count) at each data collection point. (f–i) Common within‐individual association between *KCNJ2‐AS1* (f), *KCNN2* (g), *KCNN3* (h) and *SCN4A* (i) mRNA expression levels and mean MU CV across the data collection points. Participants are represented by different colours. Abbreviations: AR21, after 21 days of active recovery; LS0, day 0 of limb suspension; LS10, after 10 days of unilateral lower‐limb suspension; MU CV, motor unit conduction velocity. Significance levels are as follows: *p < 0.05, **p < 0.01 and ***p < 0.001.

The feature importance analysis conducted including ion channel mRNA expression and muscle fibre diameters at all data collection points revealed that the top four features (i.e., the ion channels *KCNJ2‐AS1*, *KCNN2*, *KCNN3* and *SCN4A*) accounted for 49% of the predictive importance for MU CV. Notably, potassium channels exhibited a dominant influence, with the first three channels together contributing to 40% of the overall importance. A comprehensive overview of all features and their respective importance scores is presented in Figure 4a.

The changes in the mRNA expression levels (normalized reads count) of the top four ion channel genes across data collection points has also been investigated. For the mRNA expression level of *KCNJ2‐AS1* (Figure 4b), a main effect of time was observed (*p *= 0.0003, η_p_^2 ^= 0.6927). *KCNJ2‐AS1* increased at LS10 [from 533.37 (183.72) at LS0 to 748.98 (277.31) at LS10, *p *= 0.0401] and decreased at AR21 [to 373.27 (124.45), *p *= 0.0032 for LS10 vs. AR21 and *p *= 0.0401 for LS0 vs. AR21]. For the mRNA expression level of *KCNN2* (Figure 4c), a main effect of time was observed (*p *= 0.0016, η_p_^2 ^= 0.6014). *KCNN2* decreased at LS10 [from 551.70 (115.16) at LS0 to 246.54 (180.34) at LS10, *p *= 0.0090] and returned to baseline at AR21 [to 503.66 (97.62), *p *= 0.0462 for LS10 vs. AR21]. For the mRNA expression level of *KCNN3* (Figure 4d), a main effect of time was observed (*p *= 0.0137, η_p_^2 ^= 0.4581). *KCNN3* decreased at AR21 [from 5106.52 (1670.60) at LS10 to 3115.98 (746.13) at AR21, *p *= 0.0414]. For the mRNA expression level of *SCN4A* (Figure 4e), a main effect of time was observed (*p *= 0.0001, η_p_^2 ^= 0.7376). *SCN4A* increased at LS10 [from 32691.57 (5322.64) at LS0 to 37000.38 (8630.74) at LS10, *p *= 0.0208] and returned to baseline at AR21 [to 29512.52 (4981.95), *p *= 0.0003 for LS10 vs. AR21].

Changes in the mRNA expression levels of all four top‐ranked ion channel genes showed significant correlations with changes in mean MU CV throughout the intervention, as demonstrated by repeated‐measures correlation analyses (*r* = −0.60, *p *= 0.0048 for *KCNJ2‐AS1*; *r *= 0.66, *p *= 0.0014 for *KCNN2*; *r* = −0.67, *p *= 0.0011 for *KCNN3*; and *r* = −0.48, *p *= 0.0331 for *SCN4A*; Figure 4f–i).

**FIGURE 5:**
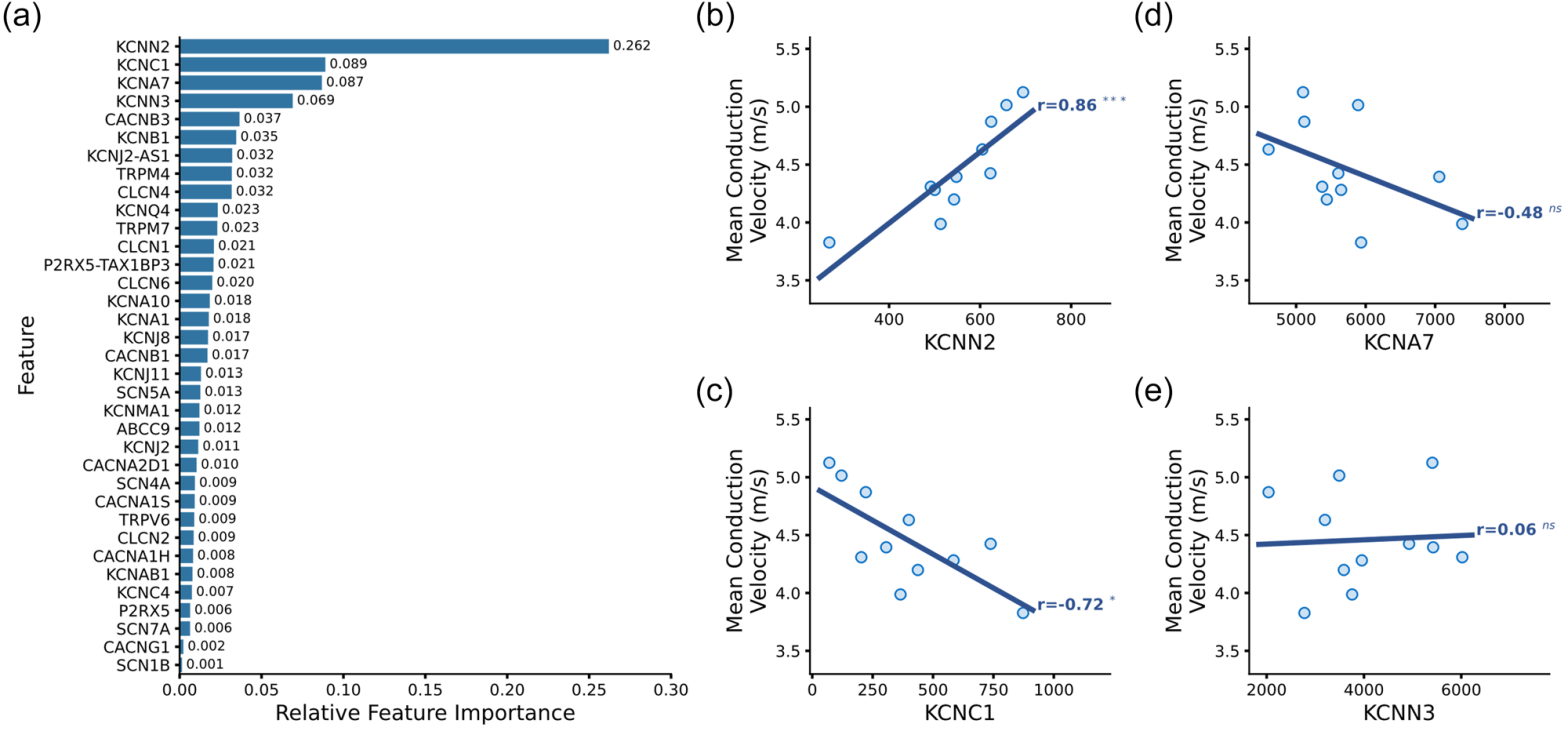
Analysis of ion channel mRNA expression levels and their association with motor unit conduction velocity (MU CV) at day 0 of limb suspension (LS0, baseline). (a) Bar plot representing the relative importance of each feature (ion channel genes) for the prediction of MU CV. (b–e) Scatter plots showing the correlation between *KCNN2* (b), *KCNC1* (c), *KCNA7* (d) and *KCNN3* (e) mRNA expression levels (normalized reads count) and mean MU CV. Significance levels are as follows: *^ns^p* > 0.05, *p < 0.05 and ***p < 0.001.

The feature importance analysis conducted including ion channel mRNA expression only at LS0 revealed that the top four ion channels (*KCNN2*, *KCNC1*, *KCNA7* and *KCNN3*, ordered for importance) accounted for 51% of the predictive importance for MU CV. An overview of all features and their respective importance scores is presented in Figure 5a.

*KCNN2* and *KCNC1* mRNA expression levels were correlated with mean MU CV at LS0 (*r* = 0.86, *p *= 0.0007 for *KCNN2*; and *r* = 0.72, *p *= 0.0129 for *KCNC1*), whereas *KCNA7* and *KCNN3* did not show significant correlations (Figure 5b–e).

## 4 DISCUSSION

### 4.1 Effect of the interventions on motor unit conduction velocity

ULLS induced a decline in MU CV, with values at LS10 being significantly lower than those at LS0 (Figure 2a). This finding is consistent with previous observations by Cescon and Gazzoni (2010), who reported similar results after 14 days of bed rest. Notably, this decline was consistent across all contraction intensities, indicating that the impairment of MU CV affects all MUs uniformly. This finding suggests a generalized decline in the MU function after 10 days of ULLS, impacting both slower and faster MUs equally. These observations align with our previous hypothesis that short‐term disuse would impair the function of all muscle units, regardless of their RT, while selectively reducing the discharge rate of lower‐threshold MUs (Valli et al., 2024b). This was initially supported by literature showing that short periods of bed rest and ULLS impair muscle fibre contractility by altering intracellular calcium handling (Monti et al., 2021) and reducing fibre‐specific force (Brocca et al., 2015) in both slow‐ and fast‐twitch muscle fibres. Overall, these findings offer additional support to our previous hypothesis that changes in the neural drive to muscle are likely to precede the metabolic adaptations associated with muscle fibre‐type shifts (Valli et al., 2024b), such as the transition from slow‐ to fast‐twitch fibres, which have been reported after prolonged periods of muscle disuse (Ciciliot et al., 2013; Hortobágyi et al., 2000).

The disuse‐induced reduction in MU CV observed at LS10 was fully reversed by AR21, with MU CV surpassing the baseline levels (Figure 2a). Given that MU CV changed throughout the intervention period, we examined whether the interventions could also induce changes in the relationship between MU RT and CV (Arendt‐Nielsen et al., 1984). At LS0, MU CV values were positively correlated with RT, whereby higher RT values corresponded to higher MU CV. This relationship remained stable throughout the intervention period, as evidenced by the similar slopes of the regression lines at each time point (Figure 2b), suggesting that the underlying physiological mechanisms linking MU CV and RT were unaffected by ULLS or AR.

Lastly, we observed that changes in MU CV were correlated with changes in MVC (Figure 2c), indicating that reductions or increases in MU CV are linked to corresponding declines or improvements in muscle force (or vice versa). This relationship underscores the importance of MU CV as an indicator of muscle function and highlights that understanding the mechanisms governing MU CV could provide new insights into the mechanisms responsible for the loss and recovery of muscle function after muscle disuse/unloading and recovery. In light of this, we investigated whether changes in structural or molecular factors, such as muscle fibre diameters and skeletal muscle ion channel mRNA expression, could be related to changes in MU CV throughout the intervention.

### 4.2 Muscle fibre diameters and their relationship to MU conduction velocity

Muscle fibre diameters, whether categorized as slow‐twitch, fast‐twitch or combined, showed no significant change across any data collection points (Figure 3a–c). This stability in muscle fibre size following short‐term disuse is consistent with previous findings from immobilization and bed rest studies, which often report limited changes in muscle fibre size (Hespel et al., 2001; Monti et al., 2021; Pišot et al., 2016; Reidy et al., 2018), either owing to a real absence of morphological change or because changes were too subtle to be detected (Reidy et al., 2018). However, when disuse models involve more severe conditions, such as cast immobilization or dry immersion, muscle fibre atrophy can be evident even within short time frames (i.e., 3–7 days; Demangel et al., 2017; Suetta et al., 2012; Wall et al., 2014). It should be noted that longer periods of disuse consistently result in a reduction of muscle fibre size (Brocca et al., 2015; Campbell et al., 2013), emphasizing that changes in muscle fibre size depend on both the severity and the duration of the disuse model. Therefore, given the relatively short (10 day) period of ULLS in the present study, the lack of detectable changes in muscle fibre diameters that we observed is consistent with the literature.

In this manuscript, muscle fibre size was assessed using the minimum Feret diameter, a metric minimally affected by sectioning procedures owing to its robustness against elongated or para‐longitudinally oriented fibres, which are commonly observed in human muscle biopsies (Briguet et al., 2004). Skeletal muscle biopsies from humans are often difficult to orient, particularly when taken from pennate muscles. This frequently results in oblique cuts, leading to kinked or distorted fibres (Dubowitz et al., 2013), which can compromise the accuracy of measurements such as perimeter or cross‐sectional area. In contrast, the minimum Feret diameter is less sensitive to fibre orientation and sectioning angle, making it a more reliable parameter in these conditions (Briguet et al., 2004). It is noteworthy that similar findings were reported by Franchi et al. (2025) in the same experimental cohort when analysing muscle fibre cross‐sectional area by immunofluorescence staining, showing no significant changes for fast‐twitch fibres and only a modest reduction in slow‐twitch fibres following active recovery (a trend also visible in our dataset, albeit not statistically significant). These consistent findings confirm that our results are robust and not much dependent on the size metric selected. Based on the findings by Franchi et al. (2025), it is also worth mentioning that fibre diameters should not be compared with whole‐muscle fibre size when looking at MU CV. The propagation of action potentials depends on the electrical properties of the sarcolemma and not on the surrounding tissues, which can be affected greatly in their volume by changes in fluid content (Franchi et al., 2025).

With the aim of identifying the factors regulating MU CV, we tested whether MU CV was correlated with muscle fibre diameters, both at baseline and across data collection points. Previous studies observed a clear correlation between CV of electrically evoked action potentials and muscle fibre size in humans (Blijham et al., 2006; Methenitis et al., 2016). Interestingly, we found no correlation between MU CV and muscle fibre diameters, both within and across data collection points.

We hypothesize that the absence of correlation observed at baseline could be attributable, in parti, to fact that we studied the CV of action potentials generated by voluntarily activated Mus, whereas Blijham et al. (2006) and Methenitis et al. (2016) studied the CV of electrically evoked action potentials. As a matter of fact, CV values vary depending on whether action potentials are generated via electrical stimulation or voluntary contraction, with the latter typically being faster and more variable (Buchthal et al., 1955). Furthermore, during electrical stimulation of the muscle, only fibres located close to the electrode are activated. These fibres tend to have nearly identical membrane potentials, resulting in a highly uniform biophysical response (Buchthal et al., 1955). Indeed, the stimulated fibres are activated because of spatial and electrical properties, without any guarantee of belonging to the same MU (Arendt‐Nielsen & Zwarts, 1989).

Therefore, although muscle fibre diameter is probably an important factor in determining muscle fibre CV, the relationship appears more complex, particularly in the context of voluntarily activated MUs, which involve multiple, spatially distributed fibres and might not directly reflect the same properties observed during electrical stimulation. To our knowledge, the only study reporting a direct comparison between voluntary muscle fibre CV and muscle fibre diameters aligns with our findings, because it found no significant correlation between the two parameters in the vastus lateralis muscle (Sadoyama et al., 1988).

Across the interventions, it could be expected that changes in MU CV do not relate to changes in muscle fibre diameters. Even acute metabolic modifications, such as the increased buffering capacity provoked by sodium bicarbonate ingestion (Hunter et al., 2009) or muscle fatigue (Vila‐Chã et al., 2012), can induce substantial alterations in CV without affecting muscle fibre diameters. Likewise, a mere 2 weeks of high‐intensity interval training or endurance exercise can cause substantial MU CV changes, yet such a short intervention is unlikely to induce measurable changes in fibre diameters (Martinez‐Valdes et al., 2018).

In conclusion, the findings of our study, together with the existing literature, show that MU CV and its changes, at least in the vastus lateralis muscle, are not correlated with muscle fibre size. This finding implies the presence of other factors with a significant role in determining MU CV and its changes.

### 4.3 Ion channels and their relationship to MU CV

The generation and propagation of action potentials in muscle fibres are regulated by ion flux across the membrane (Jurkat‐Rott & Lehmann‐Horn, 2004). The depolarization phase is initiated by the opening of voltage‐gated sodium channels, allowing Na⁺ ions to flow into the cell, leading to a rapid change in electrical charge of the membrane. The subsequent repolarization phase is driven by the opening of potassium channels, which facilitate the efflux of K⁺ ions, restoring the negative charge of the membrane. Eventually, after the conclusion of the action potential, the Na⁺/K⁺‐ATPase pump restores ion gradients by pumping Na⁺ out and K⁺ into the cell, preparing the muscle fibre for the next action potential (Feher, 2017). Maintaining an optimal balance of Na⁺ and K⁺ ions is essential for muscle excitability and contractility (Nielsen & Clausen, 2000), and the structures involved can undergo both structural and functional adaptations in response to different physical demands (Green et al., 2004).

Given the role of skeletal muscle ion channels in the generation and propagation of action potentials, we conducted a feature importance analysis to assess the predictive significance of the mRNA expression levels of 35 ion channels in explaining MU CV across the interventions (Figure 4a). Muscle fibre diameters were also included in this analysis as a reference in order to compare their contribution. The results of the feature importance analysis highlighted four key ion channel genes (*KCNJ2‐AS1*, *KCNN2*, *KCNN3* and *SCN4A*) as the most influential for the prediction of MU CV, accounting for 49% of the predictive importance of the model. Notably, the first three genes, responsible for 40% of the importance, encode potassium channels, whereas *SCN4A* encodes a sodium channel. In contrast, muscle fibre diameters contributed marginally, with only 4.4% of the predictive importance. Interestingly, the mRNA expression level of these key ion channel genes was modulated by the interventions (Figure 4b–e) and correlated significantly with MU CV values (Figure 4f–i). This differential regulation is likely to reflect an adaptation of ion channels to altered neuromuscular activity, as previously reported in animal models (Desaphy et al., 2001). The reduced neural drive and mechanical loading during disuse might drive the downregulation of genes supporting normal excitability, whereas the reintroduction of neural and mechanical stimuli during AR could promote transcriptional shifts favouring enhanced excitability, thereby facilitating restoration of neuromuscular function. These new findings highlight the importance of potassium and sodium channels in the modulation of MU CV and suggest that ion channels might have a far more crucial role in regulating MU CV than muscle fibre size, at least in our dataset.

The top‐ranked ion channel gene, *KCNJ2‐AS1*, encodes an antisense RNA of *KCNJ2*. This antisense RNA might play a regulatory role in the expression or activity of *KCNJ2*, which encodes the Kir2.1 inwardly rectifying K^+^ channel (Hibino et al., 2010). Kir2.1 contributes to the establishment of highly negative membrane potential and long‐lasting hyperpolarization, crucial for rapid Na^+^ channel activation during depolarization, which is central to action potential initiation and propagation (Hibino et al., 2010). Alterations in Kir2.1 function are implicated in Andersen's syndrome, in which dysregulation of K^+^ rectification disrupts Na^+^ channel dynamics, impairing both action potential generation and propagation, eventually resulting in periodic paralysis (Plaster et al., 2001). Furthermore, Kir2.1 has a key role in regulation of the differentiation and fusion of myoblasts to form a multinucleated muscle fibre, with mutations in the gene resulting in muscle weakness (Fischer‐Lougheed et al., 2001; Konig et al., 2004). Similar consequences are also observed for mutations in the sodium channel Na_v_1.4, which is encoded by *SCN4A* (Ptácek et al., 1991).

To understand whether ion channel mRNA expression is also correlated with MU CV in physiological conditions at baseline, we repeated the feature importance analysis including ion channel genes only at LS0 (Figure 5a). Indeed, transcriptional changes observed after interventions might not directly reflect the pathways modulating CV in a resting, unaltered state. Interestingly, although the specific gene rankings differed when considering only LS0, potassium channel genes continued to dominate in importance. The top four genes (*KCNN2*, *KCNC1*, *KCNA7* and *KCNN3*) accounted for 51% of the predictive power for MU CV at LS0. Among these, *KCNN2* showed a particularly strong correlation with MU CV values (*r* = 0.86; Figure 5b), suggesting that this gene plays a crucial role in MU CV regulation both at rest and during the intervention phases.

*KCNN2* belongs to the KCNN family, which includes *KCNN1*, *KCNN2* and *KCNN3*, encoding the small conductance calcium‐activated potassium (SK) channels KCa2.1, KCa2.2 and KCa2.3, respectively (Köhler et al., 1996). Although KCa2.1 is usually not expressed in muscles (Chen et al., 2004), KCa2.2 and KCa2.3 channels are activated by increases in intracellular Ca^2+^ and regulate the after‐hyperpolarization phase of the action potential, both in neurons and in muscle fibres (Guéguinou et al., 2014; Rahman et al., 2023). Although these ion channels are little studied in humans, it has been observed that in the rat soleus muscle both the KCa2.3 protein and *KCNN3* mRNA expression are upregulated upon denervation (Favero et al., 2008), Interestingly, early indications of denervation were also observed in our cohort at LS10 (Sarto et al., 2022), with a concomitant increase in *KCNN3* mRNA expression (Figure 4d).

In neurons, changes in the duration of the after‐hyperpolarization phase have been correlated with changes in CV, with longer after‐hyperpolarization periods usually associated with lower CV values (Cross & Robertson, 2016; Gardiner & Kernell, 1990). These findings align well with simulation studies in human muscles showing that muscle fibre CV can be modulated by altering the after‐hyperpolarization phase through changes in the activity of KCa channels (Fortune & Lowery, 2011). Given that the Ca^2+^ activating the KCa channels in skeletal muscle is released from the sarcoplasmic reticulum (Neelands et al., 2001), alterations in Ca^2+^ release could affect the activity of KCa channels and, consequently, MU CV. Evidence has shown that 10 days of bed rest reduces the Ca^2+^ content in the sarcoplasmic reticulum and diminishes the responsiveness of calcium release channels (Monti et al., 2021). This suggests that reductions in Ca^2+^ availability, attributable to sarcoplasmic reticulum dysfunction, could impair KCa channel function and modulate MU CV during periods of muscle disuse.

In summary, our findings suggest that Kir2.1 might modulate MU CV by influencing the generation of action potentials, whereas KCa channels affect the after‐hyperpolarization phase. Furthermore, the functioning of KCa channels suggests a dynamic relationship between calcium‐handling mechanisms, KCa channel activity and MU CV. The modulation of the after‐hyperpolarization phase by KCa channels in response to intracellular Ca^2+^ changes offers new insights into how ion channels might regulate MU CV beyond structural determinants.

### 4.4 Methodological considerations

Limitations in study design, HDsEMG recordings and muscle sampling have already been discussed by Sarto et al. (2022) and Valli et al. (2024b).

To ensure the most accurate estimation of MU CV in the present study, several precautions have been taken. First, the electrode grid was aligned carefully along the muscle fibre direction, identified objectively through B‐mode ultrasound imaging. Second, the estimation of MU CV was performed using state‐of‐the‐art algorithms (Farina & Merletti, 2004; Farina et al., 2002), and channel selection was executed with rigorous quality criteria, as indicated by the high XCC values obtained (Valli et al., 2024a). However, the architecture of the vastus lateralis muscle, with its pennate structure and multi‐angled fibre arrangement, might introduce some variability in MU CV estimation in comparison to fusiform muscles, such as the biceps brachii (Casolo et al., 2023). Therefore, this anatomical variability should be considered when comparing MU CV findings across studies that focus on different muscle groups, because these structural differences might influence MU CV estimates.

Muscle biopsies and HDsEMG recordings were not obtained from exactly the same region of the muscle, because the biopsy‐induced swelling and scar tissue would compromise HDsEMG recording. However, given that both assessments were performed within the same muscle (a few centimetres apart) and given the diffuse nature of the expected adaptations, it is unlikely that localized changes alone could affect correlations between molecular and electrophysiological parameters.

Although no changes in muscle fibre size were observed, it is possible that early adaptations had begun (Reidy et al., 2018) but, given the relatively short duration of the interventions, such changes might not yet have reached a magnitude detectable with biopsy techniques. Therefore, studies conducted over longer time frames could provide further insight into the relationship between changes in MU CV and muscle fibre size.

Although mRNA expression analysis provides valuable insights into rapidly changing molecular processes, it might not fully reflect the quantity and activity of the corresponding proteins. This discrepancy arises owing to the complex regulatory mechanisms that occur post‐transcriptionally, which include protein translation, degradation and post‐translational modifications, all of which determine the final protein content and functionality. Proteins often undergo modifications (e.g., phosphorylation, glycosylation) that can alter their activity without changes in mRNA expression. These modifications are essential in functional regulation, particularly in ion channels, where changes in the phosphorylation state can impact channel opening, closing and sensitivity to ions (Jurkat‐Rott & Lehmann‐Horn, 2004). Additionally, the localization of these proteins within muscle cells can influence their accessibility and function during muscle activation, as seen in various ion channels whose distribution affects muscle fibre excitability (Tricarico et al., 1997). Therefore, to reinforce and deepen the findings of this study, future investigations should focus on quantifying the protein content and activity levels, assessing post‐translational modifications and determining the spatial distribution within muscle fibres.

Ultimately, although our study identified associations between certain ion channel genes and MU CV, much remains unknown about the specific roles that these genes play in skeletal muscle and their practical influence on MU CV modulation. Therefore, further research focusing on the individual contributions of these ion channels in muscle tissue is essential to contextualise our findings fully and to clarify their impact on MU CV.

## 5 CONCLUSION

The aim of this study was to investigate the role of changes in MU CV in muscle dysfunction following disuse and active recovery, in addition to the potential biological factors underlying these changes. MU CV was significantly reduced following 10 days of ULLS in young, healthy males. Remarkably, MU CV recovered fully, and even exceeded baseline levels, following 21 days of active recovery based on resistance exercise. The observed changes in MU CV were correlated with alterations in MVC, suggesting that the reduction in MU CV might be a determinant of the loss of muscle function during periods of unloading or disuse. Although no correlation was observed between MU CV and muscle fibre diameters, mRNA expression levels of a small subset of skeletal muscle ion channel genes, particularly those linked to K^+^ transport, accounted for ∼50% in MU CV predictability both at baseline and throughout the intervention. Although the role of ion channels in action potential generation and propagation is well established, the present study is the first to relate specific ion channel mRNA expression levels directly to MU CV in humans, suggesting that ion channels might play a pivotal role in determining MU CV at baseline and its changes throughout the interventions, beyond the influence of muscle fibre size.

## AUTHOR CONTRIBUTIONS

Giacomo Valli, Fabio Sarto, Martino V. Franchi, Marco V. Narici and Giuseppe De Vito conceived and designed research. Giacomo Valli, Fabio Sarto, Elena Monti, Giuseppe Sirago, Matteo Paganini and Andrea Casolo performed experiments. Giacomo Valli, Fabio Sarto, Francesco Negro, Elena Monti, Sandra Zampieri and Julián Candia analysed data. Giacomo Valli, Fabio Sarto, Francesco Negro, Elena Monti, Sandra Zampieri, Andrea Casolo, Luigi Ferrucci and Giuseppe De Vito interpreted results of experiments. Giacomo Valli prepared figures and drafted the manuscript. All authors edited and revised the manuscript. All the authors approved the final version of the manuscript and agree to be accountable for all aspects of the work in ensuring that questions related to the accuracy or integrity of any part of the work are appropriately investigated and resolved. All persons designated as authors qualify for authorship, and all those who qualify for authorship are listed.

## CONFLICT OF INTEREST

All authors declare that there are no relationships or activities and no competing interests that might bias, or be perceived to bias, their work.

## Data Availability

RNA‐Seq datasets are available at the Gene Expression Omnibus repository: https://www.ncbi.nlm.nih.gov/geo/query/acc.cgi?acc=GSE211204. The other data supporting the findings of this study will be made available by the corresponding author upon reasonable request.
